# A Descriptive Analysis of Human Rabies in Mainland China, 2005–2020

**DOI:** 10.3390/ijerph20010380

**Published:** 2022-12-26

**Authors:** Yujuan Yue, Qiulan Chen, Di Mu, Yu Li, Wenwu Yin

**Affiliations:** 1 State Key Laboratory of Infectious Disease Prevention and Control, National Institute for Communicable Disease Control and Prevention, Chinese Center for Disease Control and Prevention, Beijing 102206, China; 2 Chinese Field Epidemiology Training Program, Chinese Center for Disease Control and Prevention, Beijing 102206, China; 3 Division of Infectious Disease Management, Chinese Center for Disease Control and Prevention, Beijing 102206, China

**Keywords:** time-series analyses, spatial analyses, demographic analyses, human rabies

## Abstract

Epidemiological characteristics of human rabies in mainland China, 2005–2020 were analyzed to evaluate the effect of rabies control in China in recent years. A total of 24,319 human rabies cases were recorded in 2097 counties in 321 cities of 31 provinces in mainland China. Only 202 cases, located in 143 counties, were recorded in 2020, compared with 3305 cases in 992 counties in 2007; however, rabies was still relatively severe in Hunan Province even in 2020. Peak periods occurred in July–November; August was often the month with the most cases. Guizhou, Hunan, Guangdong, and Guangxi Provinces, in the central and southern regions, accounted for 50.0% of the cases in 2005–2020. Cases occurred almost exclusively in rural areas with 96.7% versus 3.3% in urban areas. A paradoxical relative expansion from southern, eastern, and central towards southwestern, northwestern, northern, and northeastern regions was observed along with the overall reduction of cases. Some regions witnessed complete elimination. The male-to-female ratio was 2.33:1; 66.8% of all cases were reported in the 0–10 (13.8%) and 41–70 (53.0%) age groups. Farmers (68.3%), followed by students (12.2), and diaspora children (6.5%) were most frequently involved. Our results provide objective information for the improvement of rabies prevention and control efforts. This will aid policymakers in China and elsewhere achieve the “Zero human deaths from dog-mediated rabies by 2030” global goal.

## 1. Introduction

Rabies, caused by Species *Lyssavirus rabies*, Genus *Lyssavirus*, Family *Rhabdoviridae*, is an ancient zoonotic disease first recorded over 4000 years ago [1,2,3]. Vaccinating dogs is the most cost-effective strategy for preventing rabies in people, and human rabies vaccines exist for pre-exposure immunization [2]. Although a number of carnivore and bat species serve as natural reservoirs, dogs are the main source of human rabies deaths, contributing up to 99.0% of all rabies transmission to humans [3]. Rabies still affects over 150 countries and regions and causes about 59,000 annual human deaths worldwide, with about 40.0% of deaths occurring in individuals under 15 years old, and 95.0% occurring in developing countries in Asia and Africa [4,5,6,7]. Rabies also causes a heavy economic burden to families and society as a whole, causing estimated annual losses as high as 8.6 billion USD worldwide [4]. India ranks first in terms of the number of human rabies cases globally; China was also a country with a high rabies burden, with several hundred annual human rabies mortalities in the past [8,9,10]. Since the late 1990s, the People’s Republic of China has consistently reported the second-largest number of human rabies cases in the world; rabid dogs were the major source of transmission for both human and domestic animal rabies cases [11]. Rabies is a notifiable class B infectious disease in China; from 1960 to 2014, 120,913 human rabies cases were reported in mainland China [12]. In 2005, China formulated a national strategic plan for the prevention, control, and elimination of rabies. This was accomplished primarily through strengthening the registration, management, and immunization of dogs; improving the accessibility and standardization of post-exposure prophylaxis (PEP); and reinforcing the comprehensive control of rabies. Some progress has been made in rabies control. In this study, we focus on the seasonal, spatiotemporal, and demographic characteristics of human rabies in mainland China, from 2005–2020 to help plan resource allocation for rabies interventions, evaluate the effect of rabies control in China in recent years, and cater to the efforts made towards achieving the “Zero human deaths from dog-mediated rabies by 2030” goal set by World Health Organization [2].

## 2. Materials and Methods

### 2.1. Data Collection and Management

#### 2.1.1. Chinese National Notifiable Infectious Disease Reporting Information System (CNNDS)

Human rabies is a notifiable zoonotic disease in China. Attending physicians are required by law to report cases of rabies to Chinese Center for Disease Control and Prevention through CNNDS (http://www.chinacdc.cn/ (accessed on 10 June 2021 )). CNNDS was launched in 2004 and began recording human rabies cases in 2005.

#### 2.1.2. Case Definitions

Human rabies cases are classified into probable and confirmed cases, according to the diagnostic criteria issued by National Health Commission of the People’s Republic of China. According to the case definition of the World Health Organization (WHO), a probable case was defined as a patient with symptoms such as hyperactivity and hallucinations, or paralysis and coma, where the patient was licked, bit, or scratched by a dog, cat, or another mammal. A confirmed case was defined as a patient with a probable case whose laboratory tests revealed rabies virus infection detected by direct fluorescent antibody test, reverse-transcriptase polymerase chain reaction, or virus isolation testing of clinical specimens [12].

#### 2.1.3. Human Rabies Cases 

Human rabies case reports include sex, age, occupation, national code for current address, date of illness onset, and other information. Occupations include farmers (referring to the laborers who live in rural communities and engage in agricultural production), students (generally referring to those who are studying in schools and research institutions), and diaspora children (referring to infants and young children who do not attend nursery or kindergarten but are raised at home). Daily human rabies reports from 1 January 2005 to 31 December 2020 were obtained from CNNDS. 

#### 2.1.4. Demographic Data

The official demographic data of China included the sixth population census in 2010 and the seventh population census in 2020. However, the seventh population census at the county level in 2020 was not yet public. The sixth population census (1,339,720,000 in total) was obtained from the National Bureau of Statistics of China (http://www.stats.gov.cn/ (accessed on 10 October 2014)). The male-to-female ratio was 104.9:100.

#### 2.1.5. Vector Data

The vector data of Chinese administrative divisions in 2017, used for geographical mapping, were provided by CNNDS (http://www.chinacdc.cn/ (accessed on 10 June 2021)). There are four levels of administrative divisions in mainland China: province, city, county, and town or street. According to the administrative division, towns belong to rural areas and streets belong to urban areas. There are 31 provinces (or municipalities) comprising 343 cities and 2955 counties in mainland China. To perform spatiotemporal analyses, human rabies cases were aggregated according to national codes of current addresses, and then were geocoded and matched to the administrative boundaries using ArcGIS version 10.3 [13]. 

### 2.2. Data Analyses 

#### 2.2.1. Statistical Analyses

Time series and demographic analyses for human rabies cases were conducted using IBM SPSS Statistics version 24.0 (IBM Corp., Armonk, NY, USA). 

#### 2.2.2. Spatial Analyses

Spatial distribution analyses for human rabies in Supplementary Table S1 cases were conducted using Spatial Mapping in ArcGIS version 10.3. Spatial occurrence frequency is a measure of human rabies occurrence in a county over a number of years. The value for each year is 0 or 1.

Spatial clustering analyses were conducted using Kulldorff’s space-time scan statistic, realized by SaTScan version 9.3 (https://www.satscan.org (accessed on 10 October 2014)), to explore the locations of high-risk space-time clusters. The space-time scan statistic was defined by a cylindrical window with a circular (or elliptical) geographic base and height corresponding to time [14]. Retrospective space-time analysis scanning for clusters with high rates using the Space-Time Permutation model was employed by the county for monthly human rabies cases from 2005 to 2020. Circular scan windows were selected. The maximum spatial cluster size was set at 500 km. Monte Carlo simulations after 999 replications were used to evaluate the significance of spatiotemporal clusters (*p* ≤ 0.05 was considered statistically significant) [15].

Spatial expanding analyses of human rabies cases from 2005–2020 were conducted using the Global Polynomial Interpolation and Contour tool in ArcGIS version 10.3. When the power was set as three in this study, the trend surface of human rabies cases has the smallest root mean square. Contours, indicating the years of human rabies case occurrences, were drawn based on the trend surface. The slope of the trend surface represented the reciprocal spread speed of human rabies cases during 2005–2020.

Ethics Statement: No human or animal samples were included in the research presented in this article; therefore, ethical approval was not required for this research. 

## 3. Results

### 3.1. Time-Series Analyses for Human Rabies

**Figure 1:**
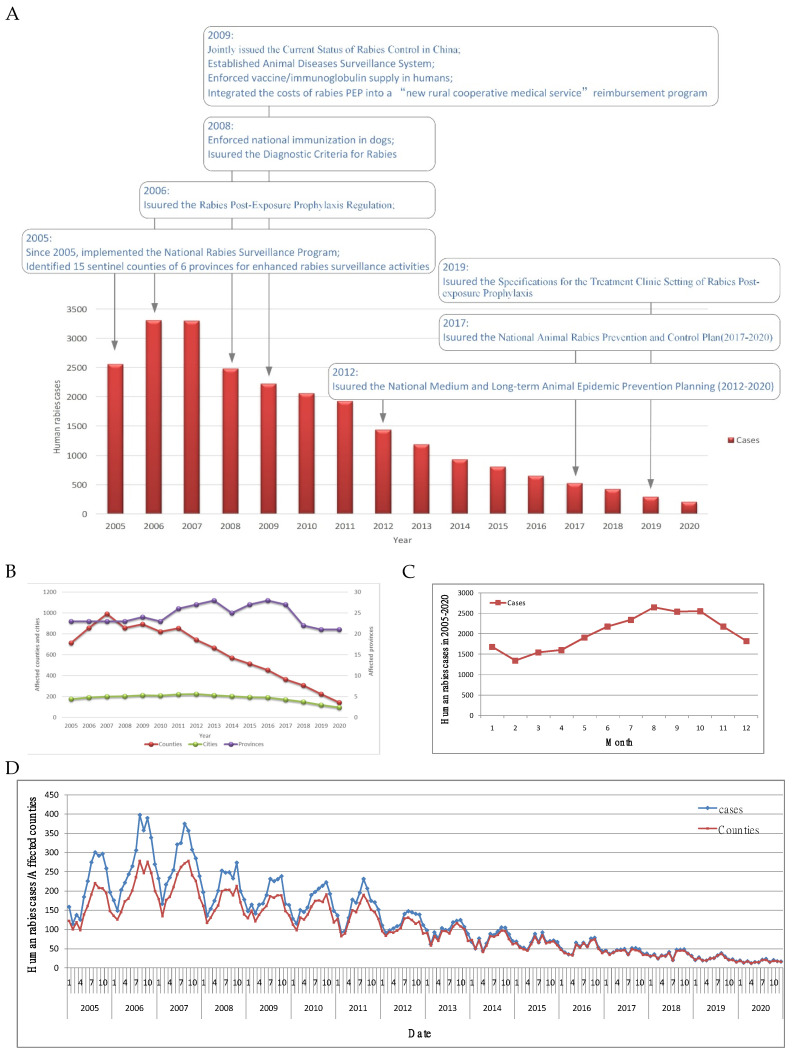
Time-series analyses of human rabies in mainland China, 2005–2020. (**A**) Yearly cases. (**B**) Affected areas. (**C**) Monthly cases in 2005–2020. (**D**) Monthly cases and affected counties.

There were 24,319 cases in mainland China, between 2005 and 2020. Of all reported cases, 98.0% were probable, and 2.0% were confirmed. Except for an upward trend from 2005 to 2007, reported cases, and counties with reported cases, decreased annually from 2007 to 2020 (Figure 1A). Affected cities and provinces remained largely unchanged, however, there was an obvious decrease in affected counties (Figure 1B). Rabies cases occurred throughout the year; however, human rabies showed a seasonal pattern, with most cases (59.3%) reported from June to November, usually peaking in August (10.9%) (Figure 1C). During these 16 years, August was the peak month in 9 years, followed by October (6 years), and September (1 year) (Figure 1D).

### 3.2. Spatial Analyses for Human Rabies

#### 3.2.1. Spatial Distribution Analyses

**Figure 2:**
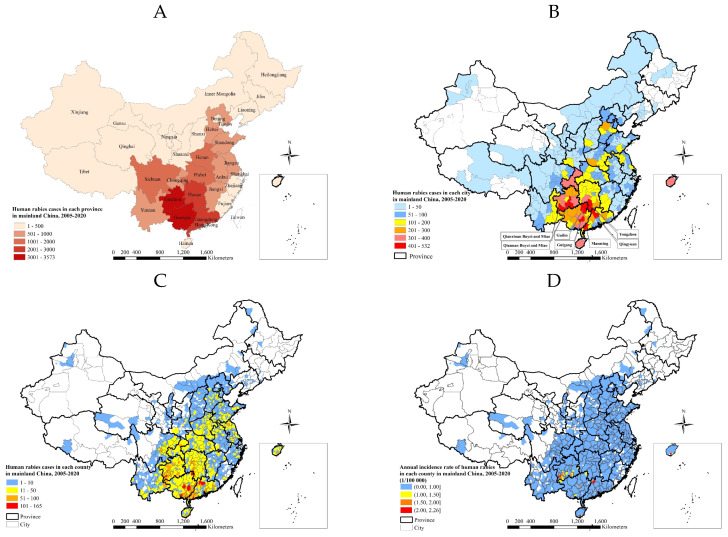
Spatial distribution of human rabies in mainland China, 2005–2020. (**A**). Cases in each province. (**B**). Cases in each city. (**C**). Cases in each county. (**D**). Incidence rate in each county.

Human rabies cases occurred in 2097 counties (70.9%), 321 cities (93.6%), and 31 provinces and municipalities (100.0%) in mainland China in 2005–2020. Geographically 3.3% (802), and 96.7% (23 517) were reported in urban and rural areas, respectively, with a ratio of 29.3:1. The four provinces with the highest number of human rabies cases, accounting for 50.0% of the total in 2005–2020, were Guizhou, Hunan, Guangdong, and Guangxi Provinces. The largest provincial concentration of cases, numbering 3573 (14.7%), was located in Guangxi Province (Figure 2A). 

The city with the highest number of cases, 532, was located in Qianxinan Buyei and Miao Autonomous Prefecture, Guizhou Province. Six cities with 400–500 reported human rabies cases were located in Guizhou, Hunan, Guangdong, and Guangxi Provinces (Figure 2B).

Counties with the most cases in 2005–2020 were distributed in the eastern regions of Guangxi Province, the western regions of Guangdong Province, and the southwestern regions of Guizhou and Hunan Province. Nine counties located in Guizhou, Guangxi, and Guangdong provinces, reported 101–165 cases. The maximum value, 165 cases, was reported in Guiping County, Guiguang City, Guangxi Province (Figure 2C). The counties with annual incidence rates greater than 2.0/100,000 were located Yangshan County in Qingyuan City, Guangdong Province (2.3/100,000), Xinren County in Qianxinan Buyei and Miao Autonomous Prefecture, Guizhou Province (2.2/100,000), and Baoting Li Autonomous Prefecture in Hainan province (2.2/100,000) (Figure 2D). There were five towns with reported cases numbering between 21 and 26 in Mainland China, from 2005–2020.

**Figure 3:**
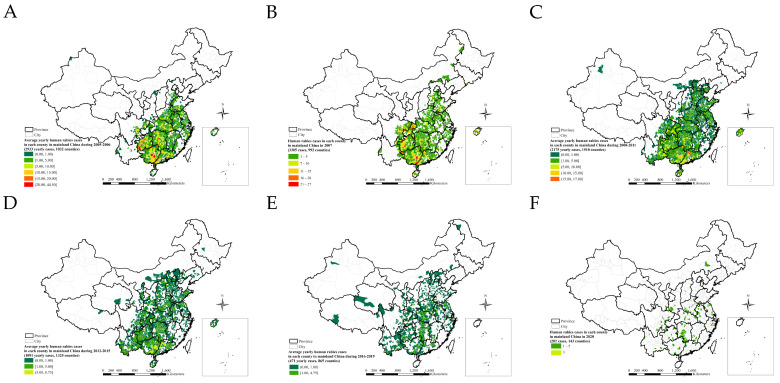
Phased spatial distribution of human rabies in mainland China, 2005–2020. (**A**) 2005–2006. (**B**) 2007. (**C**) 2008–2011. (**D**) 2012–2015. (**E**) 2016–2019. (**F**) 2020.

The time period of 2005–2020 was divided into six phases, 2005–2006, 2007 (the peak year), 2008–2011, 2012–2015, 2016–2019, and 2020 (the year with the fewest cases) (Figure 3A–F). The average annual cases decreased precipitously, and the affected counties decreased notably across these six phases. The largest number of reported cases, 3308, occurred in 855 counties in 2006; similarly, 3305 cases occurred in 992 counties in 2007. The fewest reported cases, 202, occurred in 143 counties in 2020. 

#### 3.2.2. Spatial Occurrence Frequency Analyses

**Figure 4:**
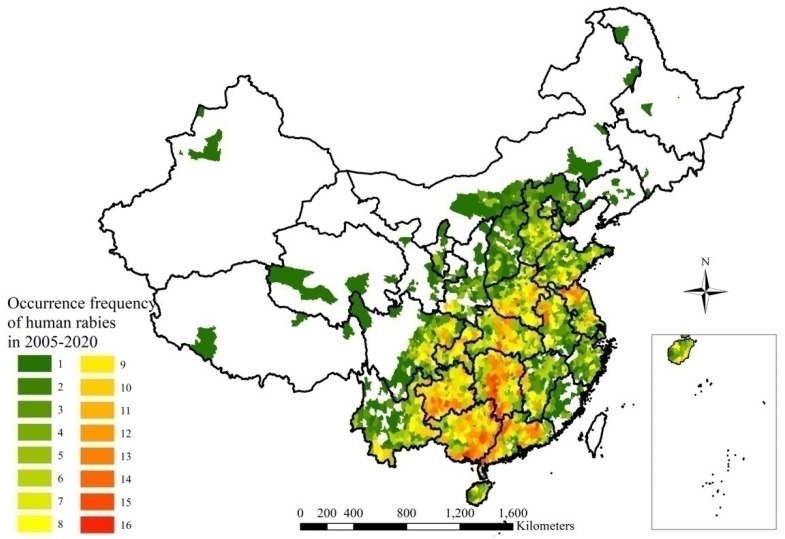
Occurrence frequency of human rabies in mainland China, 2005–2020.

High occurrences of human rabies between 13–16 times in mainland China, 2005–2020 were distributed in central and southern regions of the country, especially in Hunan, Guizhou, Guangxi, and Guangdong Provinces (Figure 4).

#### 3.2.3. Spatial Clustering Analyses

According to the time-series spatial mapping (Figure 3), cases were clustered in Guizhou, Hunan, Guangxi Provinces, and Chongqing municipalities in 2005–2006 and 2007 (Figure 3A,B); in Guangxi and Guangdong provinces in 2008–2011 and 2012–2015 (Figure 3C,D); and in Hunan Province in 2016–2019 and 2020 (Figure 3E,F)).

**Figure 5:**
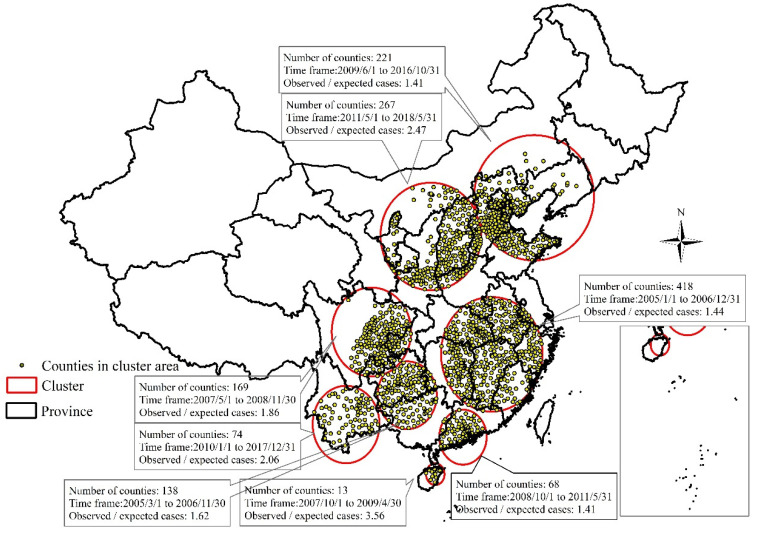
The retrospective space-time scan statistical analyses for human rabies from 2005 to 2020.

According to the results of retrospective space-time analysis scanning for human rabies, there were eight spatiotemporal clusters (Figure 5). The time spans of the spatial clusters were detected in central regions of mainland China and Guizhou province were from 2005 to 2006; in Sichuan Province from 2007 to 2008; in Yunnan Province from 2010 to 2017; in Guangdong Province from 2008 to 2011; in Hainan Province from 2007 to 2009; and in the northern and northwestern regions from 2009 to 2018. 

#### 3.2.4. Spatial Dynamics Analyses

**Figure 6:**
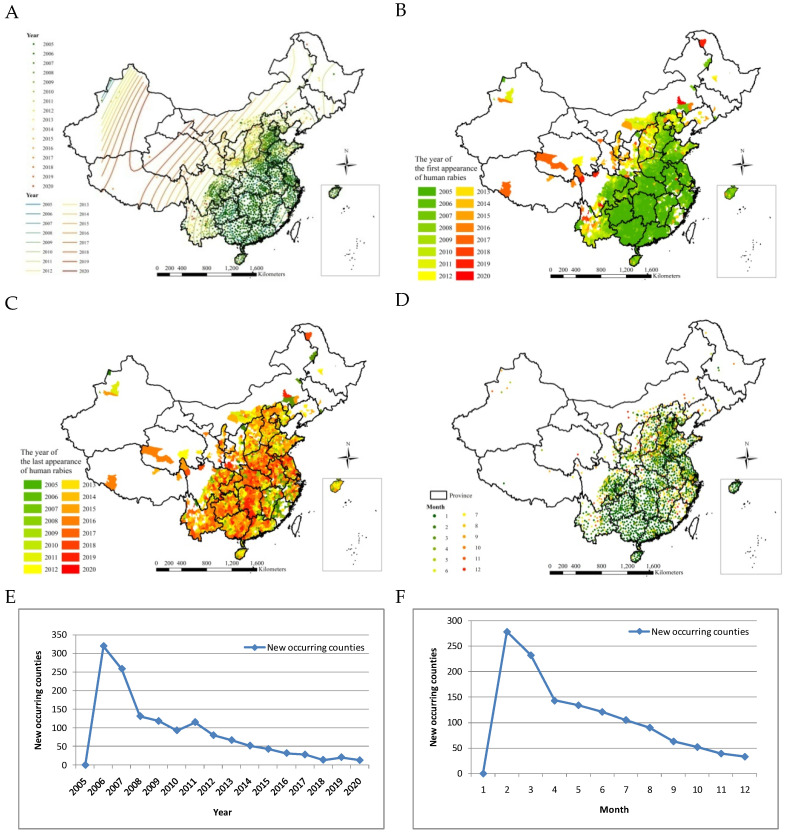
Spatial dynamics of human rabies in mainland China, 2005–2020. (**A**) Spatial expansion by year. (**B**) The year of the first appearance. (**C**) The year of the last appearance. (**D**) Spatial expansion by month. (**E**). New occurring counties by year. (**F**). New occurring counties by month.

Figure 3 and Figure 6A,B illustrate how human rabies has expanded from the southern, eastern, and central regions towards the southwestern, northwestern, northern, and northeastern regions of mainland China from 2005 to 2020. Although the number of cases did decrease, the area with frequently reported cases has paradoxically expanded, as shown in the affected provinces and cities in Figure 1C. There were 713 affected counties in 2005. Lastly, human rabies occurred in large northern, southern, and central regions from 2012–2019 (Figure 6C). No human rabies cases occurred in most of the eastern regions, such as Fujian, Jiangxi, Zhejiang, and southern Anhui, after 2011.

Human rabies has emerged from the southern and eastern regions to the regions along the southwest-northeast line and eastern regions from January to December over 2005–2020 (Figure 6D). It also shows seasonal differences in rabies outbreaks in different regions. 

Newly occurring affected counties decreased by year except 2005 (Figure 6E). Additionally, newly occurring affected counties decreased by month except January (Figure 6F).

#### 3.2.5. Human Rabies in 2020

In 2020, 202 cases occurred over 143 counties of 94 cities of 21 provinces (Figure 3F). There was only one case in each of the 106 counties (74.1% of the affected counties). The counties with seven cases each were located in Wugang County, Shaoyang City, and Ningyuan County, Yongzhou City, both in Hunan Province. The counties with four cases were located in Longhui County and Shaodong County, Shaoyang City, Hunan Province. There were eight counties with three cases each located in the southwestern regions of Hunan Province, eastern regions of Sichuan and Guizhou Province, and southeastern regions of Henan Province. A total of 29.2% of the total cases in 2020 were located in Hunan Province.

### 3.3. Demographic Analyses for Human Rabies

**Figure 7:**
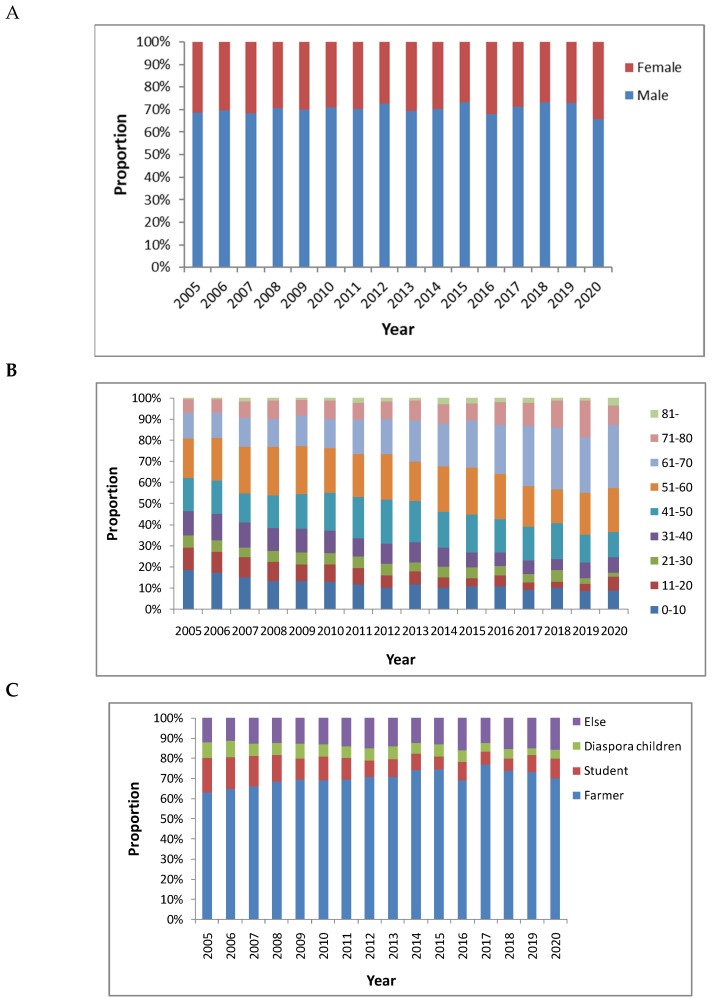
Time-series mapping of demographic characteristics for human rabies in mainland China, 2005–2020. (**A**) Gender. (**B**). Age. (**C**) Occupation.

There were 17,018 male and 7301 female cases in mainland China from 2005–2020. Cases are predominantly male, with an average male-to-female ratio of 2.33:1 (Table 1 and Figure 7A). 

The youngest reported age for a case of human rabies was five months; the oldest was 99 years. The age groups with the largest proportion of cases were 20.9% in 51–60 years old, 16.6% in 41–50 years old, 15.6% in 61–70 years old, and 13.7% in 0–10 years old (Table 1). The proportion in the 61–70 age group increased yearly (Figure 7B).

The proportion of human rabies cases was highest among farmers (68.3%), followed by students and children (Table 1). Cases in the context of occupation were relatively stable in mainland China, 2005–2020 (Figure 7C).

## 4. Discussion

A total of 24,319 human rabies cases occurred in 2097 counties of 321 cities of 31 provinces in mainland China, 2005–2020. Excluding the upward trend from 2005 to 2007, human rabies cases and affected counties have decreased annually since 2007 (Figure 1A,B). Similar conclusions were reported in previous studies. Human rabies cases increased from 2005 to 2007 and decreased from 2008 to 2011, with an accompanying change in the spatial distribution [16]. Since 2007, the incidence rate of human rabies has gradually decreased to reach 0.07/100,000 in 2014 [12]. The remarkable achievement was related closely to the Chinese government assigning increasing importance to rabies prevention and control in the 21st century. Various strategies, including surveillance, detection, vaccination, a series of laws and regulations, and cooperation between various ministries in rabies prevention and control, were adopted (Figure 1A) [17,18,19,20,21,22]. 

Human rabies occurs year-round, displaying seasonal characteristics with a peak period in July to November, as well as a peak usually during August (Figure 1C,D), which is also corroborated by the general conclusions of previous studies [12,16,23,24]. The climate in summer and autumn is mild, and dogs are active. This is also the farming season when people go out more and are more likely to come into contact with dogs, so the exposure and infection risk is higher [25].

Rabies is endemic in China. Half (50.0%) of the cases were located successively in Guizhou, Hunan, Guangdong, and Guangxi Provinces, which are in the central and southern regions of mainland China, 2005–2020 (Figure 2). A peak of cases (3573) was located in Guangxi Province. From 2004 to 2014, cases were mainly prevalent in southern regions, namely in Guangxi, Guizhou, Guangdong, and Hunan provinces, which accounted for 52% of total cases [12]. From 2016 to 2020, the top five provincial-level administrative divisions with the highest number of total cases for five years were, in decreasing order of cases, Hunan (327 cases), Henan (240 cases), Guangxi (160 cases), Guizhou (139 cases), and Hubei (138 cases), accounting for 48% of total cases reported in the nation [23]. The epidemic distribution of human rabies in China, showing a geographical distribution, is closely related to societal, economic, labor, and folk customs and culture. Human rabies is high in the south and low in the north of China, which is closely related to the density of dogs. More dogs are kept with incomplete immunization, which increases the prevalence of rabies [25]. There were seasonal differences between 2005 and 2020 in human rabies case clusters in the southern and northern regions, which was a result of climatic differences. The accumulation of human rabies cases in the southern region began relatively early, while the accumulation of human rabies cases in the northern region began later (Figure 5). Although the number of cases obviously decreased, reporting areas have paradoxically expanded from the southern, eastern, and central regions towards the southwestern, northwestern, northern, and northeastern regions of mainland China over the time period under study (Figure 6). However, some cases in northern, northeastern, and northwestern regions originated from sporadic emergent events caused by wildlife. Wildlife rabies reservoirs include foxes and raccoon dogs in northern China (Xinjiang, Inner Mongolia, Heilongjiang). In 2012, a bat lyssavirus was isolated from an insectivorous greater tube-nosed bat (*Murina leucogaster*) in Jilin Province, co-occurring with two reported bat-related human rabies cases [26]. Other wildlife in China with rabies, including rats, wolves, and hog badgers, are not the original reservoir hosts; rabies in these animals is due to unusual spill-over events from dogs or foxes, and these animals sporadically transmit rabies to humans [27]. In the eastern provinces of Jiangxi, Zhejiang, and Anhui, human cases were attributed to ferret badger bites [28]. Many regions have also recovered from human rabies, with just 143 affected counties in 2020 (Figure 3F). There were conclusions similar to those of previous studies. The rabies epidemic has continuously expanded geographically over the entire country, and new cases have been recorded in previously rabies-free and low-incidence provinces, such as the Ningxia Hui Autonomous Region, Qinghai, Gansu, Beijing, Jilin, and Tibet, in the last decade [26,29]. Although a few places have recovered from human rabies, most affected places still suffered from the disease in 2008–2011 [16]. 

The male-to-female ratio of human rabies cases in mainland China was 2.33:1. The cases in the 0–10 and 41–70 age groups accounted for 66.8% of total cases. Farmers, students, and children accounted for 68.3%, 12.2%, and 6.5%, respectively, of the total cases in mainland China in 2005–2020 (Table 1 and Figure 7). In 2012, rabies cases were mainly in farmers, students, and children, accounting for 70.9%, 8.3%, and 5.8% of total cases, respectively [30]. In 2013 the proportions of affected populations were similar [31]. In the period 2001–2014, farmers accounted for 65.0% of the total cases in mainland China, followed by students and children (24.1%) [12]. Men are more likely to work outdoors or in the field, which made them more susceptible to dog bites. In recent years, more young men in rural areas have left the area to work, while most farmers at home are middle-aged and elderly. Farmers were more easily infected with rabies, which could be related to the lack of availability of health resources, travel difficulties, and expense of PEP [8].

Through concerted stakeholder efforts, great progress has been made in the prevention and control of rabies in China. The reported cases of human rabies decreased yearly. There were 202 cases located in 143 counties in 2020, a decrease of 93.8% and 85.6% in cases and counties, respectively, when compared with the most recent peak in 2007 (3305 human cases in 992 counties). It demonstrates a sporadic trend in rabies occurrence. The year 2020 was a landmark year to drive progress toward the goal of “Zero human deaths from dog-mediated rabies by 2030.” It was closely related to the rabies policies implemented by the Chinese government, and less related to the lack of travel during the COVID-19 epidemic. The first step towards rabies elimination has been achieved, which lays the foundation for the elimination of rabies in China. This indicates that China’s rabies elimination plan has achieved a landmark victory under the successful implementation of various policies and measures. Therefore, it is completely feasible for China to achieve the goal of “Zero human deaths from dog-mediated rabies by 2030” proposed by the WHO. 

Some limitations of this study should be mentioned. Firstly, we could only obtain human rabies case reports, but not the information about canine rabies and dog vaccination coverage managed by other government departments, so we could not show a direct relationship between human and canine rabies. Secondly, we could only obtain the sixth population census and were not able to analyze the changes in incidence rates during different phases using a specific method such as a space-time Poisson model. Thirdly, the case reports used in this study lacked the sources of infection. It is recommended to add an infection source field in case reports in the future.

## 5. Conclusions

We aimed to explore the epidemiological characteristics of human rabies in mainland China, from 2005–2020, to understand the progress of rabies control and prevention. A total of 24,319 human rabies cases were recorded. The reported cases of human rabies decreased annually, which proved that the strategies and measures of China’s rabies prevention and control project were effective. In 2020, only 202 cases, located in 143 counties, were recorded, compared with 3305 cases in 992 counties in 2007; however, human rabies was still relatively severe in Hunan Province in 2020. Human rabies seasonally peaks from July to November, with most cases located in the central and southern regions of mainland China. Although the number of cases did decrease, the locations of the majority of cases paradoxically expanded from southern, eastern, and central regions towards southwestern, northwestern, northern, and northeastern regions; some regions even experienced total eradication. The male-to-female ratio overall was 2.33:1. The 0–10 and 41–70 age groups accounted for 66.8% of all cases. Farmers accounted for 68.3% of the total cases. China should continue to implement the existing proven effective rabies prevention and control strategies and measures to eliminate rabies as soon as possible.

## Figures and Tables

**Table 1:** Demographic analysis for human rabies.

	Group Division	Human Rabies Cases	Proportion (%)
**Gender**	Male	17,018	70.0
	Female	7301	30.0
**Age**	0–10	3347	13.8
	11–20	1950	8.0
	21–30	1249	5.1
	31–40	2531	10.4
	41–50	4034	16.6
	51–60	5080	20.9
	61–70	3799	15.6
	71–80	1987	8.2
	81–	342	1.4
**Occupation**	Farmer	16,622	68.3
	Student	2966	12.2
	Diaspora children	1580	6.5
	Else	3151	13.0

## Data Availability

Case reports were from CNNDS (http://www.chinacdc.cn/ (accessed on 10 June 2021)). The vector data of Chinese administrative divisions were from CNNDS (http://www.chinacdc.cn/ (accessed on 10 June 2021)). The sixth population census was from the National Bureau of Statistics of China (http://www.stats.gov.cn/ (accessed on 10 October 2014)).

## Supplementary Materials

The following supporting information can be downloaded at: https://www.mdpi.com/article/10.3390/ijerph20010380/s1, Table S1: Spatial distribution of human rabies in mainland China, 2005–2020.

## References

[1] International Committee on Nomenclature of Viruses\Taxon Details of Lyssavirus Rabies. https://ictv.global/taxonomy/taxondetails?taxnode_id=202101733.

[2] World Health Organization\Textbar About Rabies. https://www.who.int/news-room/fact-sheets/detail/rabies.

[3] Fooks A.R., Banyard A.C., Horton D.L., Johnson N., McElhinney L.M., Jackson A.C. (2014). Current status of rabies and prospects for elimination. Lancet Lond. Engl..

[4] Chen Q. (2021). Accelerate the Progress Towards Elimination of Dog-Mediated Rabies in China. China CDC Wkly..

[5] Hampson K., Coudeville L., Lembo T., Sambo M., Kieffer A., Attlan M., Barrat J., Blanton J., Briggs D., Cleaveland S. (2015). Estimating the global burden of endemic canine rabies. PLoS Negl. Trop. Dis..

[6] Meslin F.X., Briggs D.J. (2013). Eliminating canine rabies, the principal source of human infection: What will ittake?. Antivir. Res..

[7] WHO Expert Consultation on Rabies. https://apps.who.int/iris/handle/10665/85346.

[8] Yin W., Dong J., Tu C., Edwards J., Guo F., Zhou H., Yu H., Vong S. (2013). Challenges and needs for China to eliminate rabies. Infect. Dis. Poverty.

[9] Abbas S.S., Kakkar M. (2015). Rabies control in India: A need to close the gap between research and policy. Bull. World Health Organ..

[10] Bagcchi S. (2015). India fights rabies. Lancet Infect. Dis..

[11] Tu C., Feng Y., Wang Y. (2018). Animal rabies in the People’s Republic of China. Rev. Sci. Tech. Off. Int. Epiz..

[12] Zhou H., Vong S., Liu K., Li Y., Mu D., Wang L., Yin W., Yu H. (2016). Human Rabies in China, 1960–2014: A Descriptive Epidemiological Study. PLoS Negl. Trop. Dis..

[13] ESRI ArcGIS 10.3 Help.

[14] Kulldorff M. (2014). SaTScan User Guide for Version 9.3.

[15] Ling C., Gruebner O., Krämer A., Lakes T. (2014). Spatio-temporal patterns of dengue in Malaysia: Combining address and sub-district level. Geospat. Health.

[16] Guo D., Zhou H., Zou Y., Yin W., Yu H., Si Y., Li J., Zhou Y., Zhou X., Magalhaes R. (2013). Geographical Analysis of the Distribution and Spread of Human Rabies in China from 2005 to 2011. PLoS ONE.

[17] Yin W., Fu Z., Gao G. (2021). Progress and Prospects of Dog-Mediated Rabies Elimination in China. China CDC Wkly..

[18] Chinese Ministry of Health, Ministry of Public Security, Chinese Ministry of Agriculture, Chinese Food and Drug Administration (2009). Current Status of Rabies Control in China. http://www.gov.cn/gzdt/2009-09/27/content_1428014.htm.

[19] The General Office of the State Council (2012). National Medium and Long-Term Animal Epidemic Prevention Planning (2012–2020). http://www.moa.gov.cn/zwllm/ghjh/201205/t20120530_2678977.htm.

[20] Ministry of Agriculture and Rural Affairs of the Peoples Republic of China (2017). National Animal Rabies Prevention and Control Plan (2017–2020). http://www.moa.gov.cn/nybgb/2017/dlq/201712/t20171231_6133713.htm.

[21] The National Health and Family Planning Commission (2006). Rabies Post-Exposure Prophylaxis Regulation. http://www.caaa.cn/show/standard.php?ID=279.

[22] The National Health and Family Planning Commission (2016). Rabies Post-Exposure Prophylaxis Technical Guidelines. https://wenku.baidu.com/view/f55f342d30b765ce0508763231126edb6f1a76f2.html.

[23] Liu Z., Lin M., Tao X., Zhu W. (2021). Epidemic Characteristics of Human Rabies-China, 2016–2020. China CDC Wkly..

[24] Liu J., Duo L., Tao X., Zhu W. (2021). Epidemiological characteristics of human rabies in China, 2016–2018. Chin. J. Epidemiol..

[25] Tu C. (2016). Prevalence Status and Causes of Rabies in China. Spec. Rep. China Vet. Dev. Forum.

[26] Miao F., Li N., Yang J., Chen T., Liu Y., Zhang S., Hu R. (2021). Neglected challenges in the control of animal rabies in China. One Health.

[27] Wang L., Tang Q., Liang G. (2014). Rabies and rabies virus in wildlife in mainland China, 1990–2013. Int. J. Infect. Dis..

[28] Zhang S., Tang Q., Wu X., Liu Y., Zhang F., Rupprecht C., Hu R. (2009). Rabies in ferret badgers, southeastern China. Emerg. Infect. Dis..

[29] Tao X., Guo Z., Li H., Jiao W., Shen X., Zhu W., Rayner S., Tang Q. (2015). Rabies cases in the west of China have two distinct origins. PLoS Negl. Trop. Dis..

[30] Zhou H., Li Y., Mu D., Yin W., Yu H. (2015). Analysis of epidemiological features of human rabies in China. Chin. J. Epidemiol..

[31] Zhou H., Mu D., Li Y., Chen Q., Yin W., Yu H. (2015). Epidemiological features and analysis on human rabies in China. Int. J. Virol..

