# Supplementary material for: A Descriptive Analysis of Human Rabies in Mainland China, 2005–2020
Source: Int J Environ Res Public Health. 2022 Dec 26;20(1):380. doi: 10.3390/ijerph20010380 (PMC9819004; doi:10.3390/ijerph20010380)
Supplement: Supplementary file 1 [file ijerph-20-00380-s001.zip › ijerph-2113362-supplementary.pdf]

Table S1. Spatial distribution of human rabies in mainland China, 2005-2020

Administrative division	Peak range	Spatial location of peaks (Cases)
Province	>2500 cases	Guangxi (3 573),<br>Guizhou (3 036),<br>Guangdong (2 721),<br>Hunan (2 584)
City	Among 400-500 cases	Qianxinan Buyei and Miao Autonomous Prefecture, Guizhou Province (532),<br>Qiannan Buyei and Miao Autonomous Prefecture, Guizhou Province,<br>Guilin and Guigang City, Guangxi Province,<br>Yongzhou City, Hunan Province,<br>Maoming and Qingyuan City, Guangdong Province
County	Among 101-165 cases	Xinren County, Qianxinan Buyei and Miao Autonomous Prefecture, Guizhou Province,<br>Guiping and Pingnan County in Guigang City, Quanzhou County in Guilin City, Xinbing County in Laibin City, and Qinbei County in Qinzhou City in Guangxi Province,<br>Yangshan and Yingde County in Qingyuan City, and Dianbai County in Maoming City in Guangdong Province
Town	Among 21 - 26 cases	Baling Town, Xinren County, Qianxinan Buyei and Miao Autonomous Prefecture, Guizhou Province,<br>Qigong Town, Yangshan County, Qingyuan City, Guangdong Province<br>Dawan Town, Yingde County, Qingyuan City, Guangdong Province<br>Yangjiao Town, Maoguang County, Maoming City, Guangdong Province<br>Shitang Town, Quanzhou County, Guilin City, Guangxi Province
